# Arctigenin from *Fructus Arctii* (Seed of Burdock) Reinforces Intestinal Barrier Function in Caco-2 Cell Monolayers

**DOI:** 10.1155/2015/368105

**Published:** 2015-10-13

**Authors:** Hee Soon Shin, Sun Young Jung, Su Yeon Back, Jeong-Ryong Do, Dong-Hwa Shon

**Affiliations:** ^1^Korea Food Research Institute, 1201-62 Anyangpangyo-ro, Bundang-gu, Seongnam-si, Kyeonggi-do 463-746, Republic of Korea; ^2^Division of Food Biotechnology, Korea University of Science and Technology, Daejeon 305-350, Republic of Korea

## Abstract

*Fructus Arctii* is used as a traditional herbal medicine to treat inflammatory diseases in oriental countries. This study aimed to investigate effect of *F. Arctii* extract on intestinal barrier function in human intestinal epithelial Caco-2 cells and to reveal the active component of *F. Arctii*. We measured transepithelial electrical resistance (TEER) value (as an index of barrier function) and ovalbumin (OVA) permeation (as an index of permeability) to observe the changes of intestinal barrier function. The treatment of *F. Arctii* increased TEER value and decreased OVA influx on Caco-2 cell monolayers. Furthermore, we found that arctigenin as an active component of *F. Arctii* increased TEER value and reduced permeability of OVA from apical to the basolateral side but not arctiin. In the present study, we revealed that *F. Arctii* could enhance intestinal barrier function, and its active component was an arctigenin on the functionality. We expect that the arctigenin from *F. Arctii* could contribute to prevention of inflammatory, allergic, and infectious diseases by reinforcing intestinal barrier function.

## 1. Introduction

*Arctium lappa* (burdock) is a biennial plant of the* Arctium* genus in the Asteraceae family. Its root is widely used as a dietary ingredient, and its seed (called* Fructus Arctii*) is used as a traditional herbal medicine in oriental countries such as China, Japan, and Korea. In particular,* F. Arctii* has been used for the treatment of sore throat, urticaria, and furuncle. The anti-inflammatory and antioxidant properties of* F. Arctii* are supported by its ethnopharmacological use for the treatment of inflammation, wherein it inhibits proinflammatory factors such as nuclear factor kappa B (NF-*κ*B), inducible nitric oxide synthase, and oxidative stress [1, 2].

Intestinal epithelial cells regulate the influx of nutrients and the efflux of xenobiotics against substances entering the gut intestinal tract [3]. The tight junction (TJ) proteins expressed in the cells play a key role in intestinal barrier function by protecting against toxic substances, allergens, and macromolecules derived from food [4]. However, dysfunction or destruction of TJ may cause inflammatory diseases such as inflammatory bowel disease, irritable bowel syndrome, leaky gut syndrome, and food allergy [5–8]. Recently, various components derived from food and plants have been known to enhance intestinal barrier function. For example, theaflavins in black tea could enhance intestinal barrier function by increasing the expression of TJ-related proteins (occludin, claudin-1, and zonula occludens-1) through the activation of AMP-activated protein kinase in Caco-2 cells [9].

The Caco-2 cells, which are derived from colon carcinoma, spontaneously undergo the process of differentiation that leads to the formation of monolayers. The cell monolayers have been used as an* in vitro* model that mimics the human intestinal epithelium [10, 11]. In this study, we established an* in vitro* system to evaluate the effect of food- and plant-derived extracts including* F. Arctii* on intestinal barrier function in Caco-2 cell monolayers. In particular, the effect of* F. Arctii* was focused on intestinal barrier function, and the permeability was measured using ovalbumin (OVA) as one of substrates absorbed through paracellular diffusion. Furthermore, we investigated what component was an active compound in the extract by fractionation, HPLC analysis, and ability to enhance the intestinal barrier function in human intestinal epithelial Caco-2 cells.

## 2. Materials and Methods

### 2.1. Materials

The Caco-2 cell line was obtained from the American Type Culture Collection (Rockville, MD, USA). Dulbecco's modified Eagle's medium (DMEM), fetal bovine serum (FBS), penicillin-streptomycin, nonessential amino acids (NEAA), phosphate-buffered saline (PBS), and Hank's balanced salt solution (HBSS) were purchased from WelGENE (Daegu, Korea). OVA and bile salts (mixture of sodium cholate and sodium deoxycholate) were purchased from Sigma (St. Louis, MO, USA). Arctiin and arctigenin were purchased from Santa Cruz Biotechnology (Santa Cruz, CA, USA).

### 2.2. Sample Preparation

The 9 samples that were used in this study were purchased from Plant Extract Bank at the Korea Research Institute of Bioscience & Biotechnology (Daejeon, Korea) and Kyung-dong Oriental Pharmacy (Seoul, Korea) and identified by Professor Y. Bu, Department of Herbal Pharmacology, Kyung Hee University. The specimen (KFRI-SL-2170) has been kept in Korea Food Research Institute.* Fructus Arctii* (400 g) was pulverized to powder, and then the extract from the powder was obtained by reflux extraction in 95% ethanol (4 L) for 3 h twice. The ethanol extract was concentrated under vacuum in a rotary evaporator and then freeze-dried for 3 days (84 g, yield 21.1%).

### 2.3. Cell Culture

The Caco-2 cells were cultured at 37°C in humidified air containing 5% CO_2_. The cells were maintained in a 100 mm dish with DMEM containing 1000 mg/L of glucose and supplemented with 10% FBS, 1% NEAA, 100 U/mL of penicillin, and 100 *μ*g/mL of streptomycin. The cells were seeded at a density of 2 × 10^5^ cells/mL on a 12-transwell plate (Costar, Corning, NY, USA) and allowed to grow for 3 weeks for the experiments.

### 2.4. Measurement of Transepithelial Electrical Resistance

The integrity of the Caco-2 cell monolayers was checked by measuring the transepithelial electrical resistance (TEER) by using a Millicell-ERS device (Millipore, Bedford, MA, USA). The monolayers of Caco-2 cells (300–500 Ω cm^−2^) were preincubated with HBSS for 30 min at 37°C in a CO_2_ incubator to stabilize the cell monolayers. The cell monolayers were treated with each sample and bile salts for 60 min, and OVA was added for 3 h at 37°C. The presence of bile salts weakens intestinal barrier function [12]. However, the bile salts actually exist in gastrointestinal tract* in vivo*, and the addition of bile salts in experimental* in vitro* system can mimic* in vivo* system. The TEER value was measured, and OVA influx was also detected by ELISA.

### 2.5. ELISA

Coating anti-OVA primary antibodies (ab17290) and horseradish peroxidase- (HRP-) conjugated anti-OVA secondary antibodies (ab20415) for ELISA were purchased from Abcam (Cambridge, MA, USA). 96-well immunoplates were coated by overnight incubation at 4°C with coating anti-OVA primary antibodies. After blocking for 2 h with 1% bovine serum albumin, the supernatant was incubated at RT for 2 h. After washing by PBS with 0.05% Tween 20, HRP-conjugated anti-OVA secondary antibodies were added for 1 h on plates, and then each well was reacted by 3,3′,5,5′-tetramethylbenzidine substrate solution in the dark. Finally, stop solution (2 N sulfuric acid) was added, and then absorbance was measured at 450 nm using an Epoch microplate reader (BioTek, Winooski, VT, USA).

### 2.6. Solvent Fractionation of* Fructus Arctii* Extract

**Figure 1:**
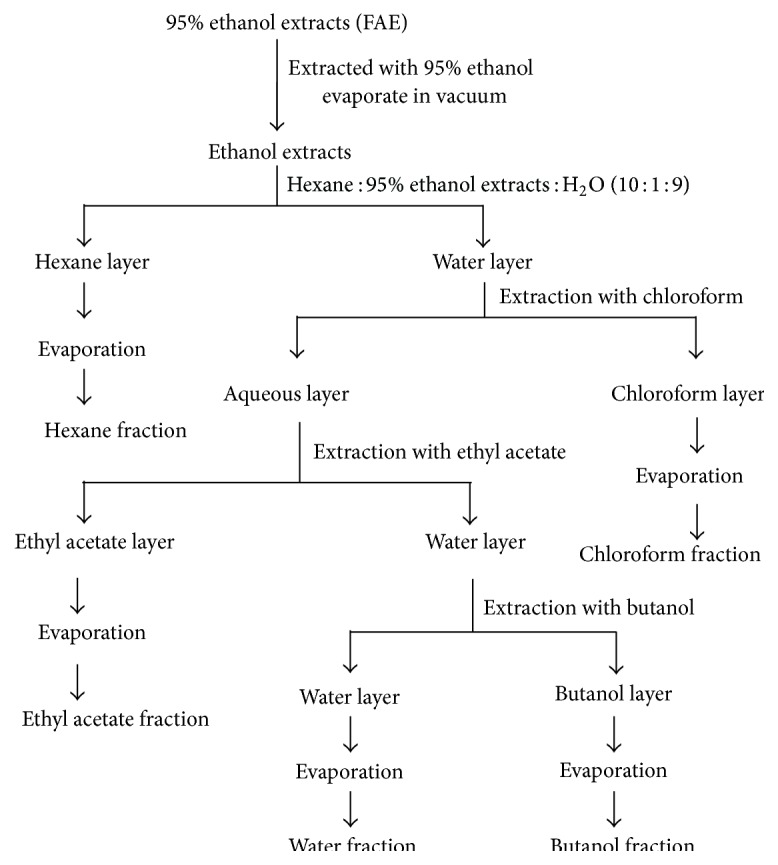
Fraction separation from ethanolic extract of* Fructus Arctii*.

The resultant ethanol extract of* Fructus Arctii* was further fractionated with different solvents. Dried* Fructus Arctii* extract (FAE) was suspended in distilled H_2_O (FAE : H_2_O, 1 : 9). And then the solution was suspended with hexane solvent (FAE in H_2_O : Hexane, 1 : 1). Each solvent (chloroform, ethyl acetate, butanol, and water) was sequentially added in FAE solution, and then each fraction was sequentially partitioned with hexane, chloroform, ethyl acetate, butanol, and water (Figure 1). All solvent fractions were evaporated until dry. The detailed method and yields of solvent fractions are shown in Table 1.

### 2.7. Determination of Total Phenolic Content

Measurement of total phenolic content (TPC) was based on the method described by Shin et al. [13]. TPC was calculated using tannic acid (Sigma-Aldrich) as a standard.

### 2.8. HPLC Analysis

High-performance liquid chromatography (HPLC) analysis was carried out with a Jasco PU-2080 plus liquid chromatography system (JASCO, Tokyo, Japan) equipped with multiwavelength detector Jasco UV-2075 plus (JASCO, Tokyo, Japan). Samples were injected to 1 *μ*g/mL by autoinjector AS-2057 plus (JASCO, Tokyo, Japan), and ODS C18 column (250 × 4.6 mm) was used to separate compounds at 40°C in column adapter CO-2060 plus (JASCO, Tokyo, Japan). Solvents were used mixture of acetonitrile (A) and water (B) in gradient mode (eluent A: 20 to 40% in 35 min), and the flow rate was 1 mL/min. UV wavelength for detection was 280 nm.

### 2.9. Statistical Analysis

Results are expressed as the mean ± standard deviation (SD). A statistical analysis was performed using the SAS statistical software package (SAS Institute, Cary, NC, USA). Differences between the experimental data were assessed by 1-way analysis of variance (ANOVA), followed by Duncan's multiple-range test; a value of *P* < 0.05 was considered significant.

## 3. Results

### 3.1. Effects of Natural Material Extracts on Intestinal Barrier Function in Monolayers of Caco-2 Cells

**Figure 2:**
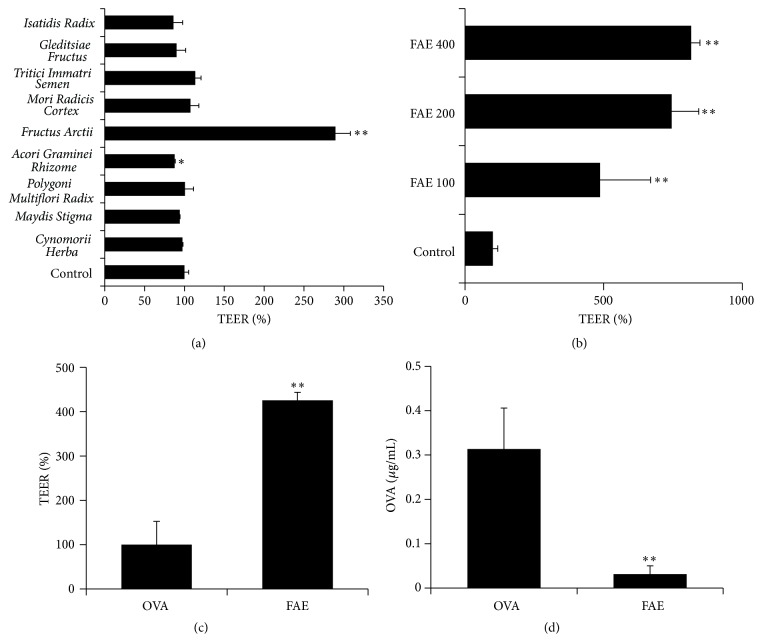
*Fructus Arctii* extract increased TEER value and decreased OVA influx in Caco-2 cells. The monolayers of Caco-2 cells were preincubated with HBSS for 30 min at 37°C in a CO_2_ incubator to stabilize the cell monolayers. The cell monolayers were treated with each sample and bile salts for 60 min, and OVA was added for 3 h at 37°C. (a) Changes of TEER were measured by treating the cell monolayers with nine extracts (400 *μ*g/mL) and (b)* Fructus Arctii* extract (FAE) at various doses (100, 200, and 400 *μ*g/mL) for 3 h. (c and d) Changes in TEER value and OVA influx induced by FAE were detected by ELISA. Each value is presented as mean ± SD (*n* = 3). Bars are significantly different from the control at ^*∗*^ *P* < 0.05 and ^*∗∗*^ *P* < 0.01. Data were analyzed using ANOVA followed by Duncan's multiple-range test.

We investigated the effects of 95% ethanolic extracts from nine natural materials derived from plants (*Cynomorii Herba*,* Maydis Stigma*,* Polygoni Multiflori Radix*,* Acori Graminei Rhizoma*,* F. Arctii*,* Mori Radicis Cortex*,* Tritici Immatri Semen*,* Gleditsiae Fructus*,  and* Isatidis Radix*) on intestinal barrier function by measuring TEER in Caco-2 cell monolayers. Among the nine natural materials, the FAE significantly increased the TEER value by 289%, compared to the control, in Caco-2 cell monolayers (Figure 2(a)). In contrast, the other eight natural material extracts had no effect on the TEER value. Furthermore, the FAE dramatically enhanced the TEER value in a dose-dependent manner (Figure 2(b)). Next, we examined whether the FAE-induced enhancement of intestinal barrier function could suppress allergen permeation via paracellular diffusion pathway. The results showed that FAE treatment reduced OVA permeation across Caco-2 cell monolayers by enhancing the intestinal barrier function (Figures 2(c) and 2(d)). These results indicated that* F. Arctii* could enhance intestinal barrier function, preventing allergens and toxic and xenobiotic substrates from entering the intestine.

### 3.2. Effects of FAE Fractions on TEER and OVA Flux in Caco-2 Cell Monolayers

**Figure 3:**
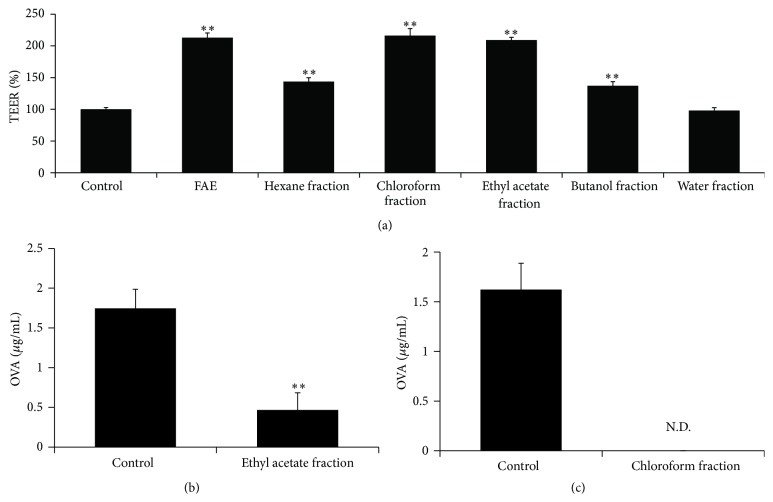
Ethyl acetate and chloroform fractions isolated from* Fructus Arctii* extract reinforced TEER and reduced OVA influx in Caco-2 cells. FAE was fractionated to five fractions, hexane, chloroform, ethyl acetate, butanol, and water fractions. Effects of fractions (200 *μ*g/mL) on TEER values were measured in the cell monolayers, and amount of permeated OVA was also detected in the basolateral side. Each value is presented as mean ± SD (*n* = 3). Bars are significantly different from the control at ^*∗∗*^ *P* < 0.01. Data were analyzed using ANOVA followed by Duncan's multiple-range test.

To investigate the active components of FAE, FAE was fractionated using different polar solvents such as hexane, chloroform, ethyl acetate, butanol, and water. Among the five fractions extracted from FAE, chloroform and ethyl acetate fractions significantly increased TEER value in Caco-2 monolayers and also inhibited the OVA permeation across Caco-2 cell monolayers (Figure 3). Although TEER values were significantly increased by hexane and butanol fractions, the increases were similar to the level of TEER value in initial control (normal condition). Therefore, we thought that the hexane and butanol fractions recovered TEER values reduced by bile salts in Caco-2 cell monolayers. These results demonstrated that the FAE-induced enhancement of intestinal barrier function was owing to compounds in chloroform and ethyl acetate fractions. Thus, we investigated TPC of the fractions to determine the properties of these components. Table 1 shows the TPC of fractions; the ethyl acetate fraction contained the highest amount of phenolic compounds (412.78 ± 10.47). Chloroform and butanol fractions also contained large quantities of phenolic compounds; however, the hexane and water fractions had relatively low content of phenolic compounds. Therefore, we suggest that the active components of chloroform and ethyl acetate fractions might be nonpolar polyphenols.

### 3.3. Effects of Single Component (Arctiin and Arctigenin) on TEER and OVA Flux in Caco-2 Cell Monolayers

**Figure 4:**
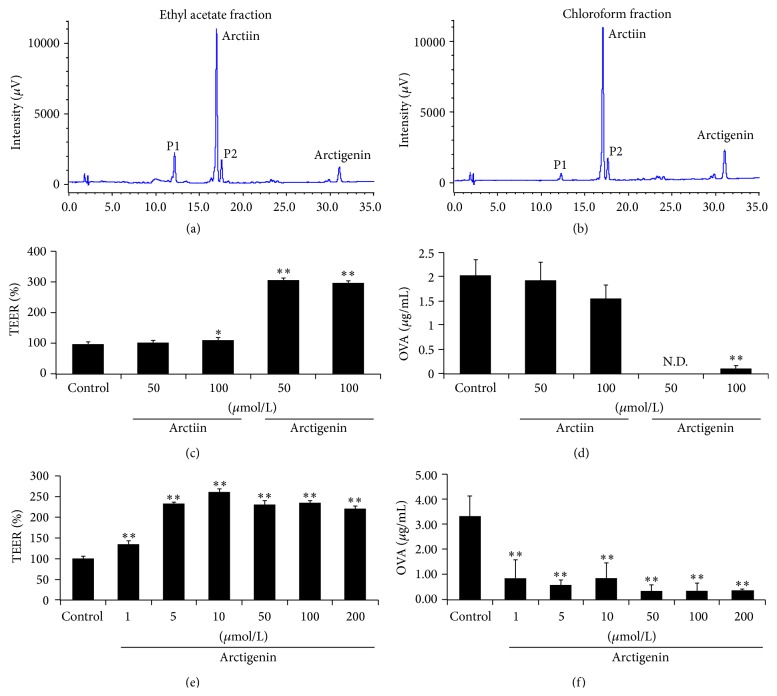
Arctigenin, arctiin, from* Fructus Arctii* extract enhanced intestinal barrier function in Caco-2 cells. (a and b) To identify active components in* Fructus Arctii* extract, HPLC analysis was carried out with a Jasco PU-2080 plus liquid chromatography system equipped with multiwavelength detector. Ethyl acetate and chloroform fractions were injected (1 *μ*g/mL each) by autoinjector, and an ODS C18 column (250 × 4.6 mm) was used to separate compounds at 40°C in column adapter. Solvents used were a mixture of acetonitrile (eluent A) and water (eluent B) in gradient mode (eluent A: 20 to 40% in 35 min), and the flow rate was 1 mL/min. UV wavelength for detection was 280 nm. (c–f) The effects of arctiin and arctigenin on TEER and OVA permeability were examined in Caco-2 cells. Each value is presented as mean ± SD (*n* = 3). Bars are significantly different from the control at ^*∗*^ *P* < 0.05 and ^*∗∗*^ *P* < 0.01. Data were analyzed using ANOVA followed by Duncan's multiple-range test.

Next, we analyzed the components in chloroform and ethyl acetate fractions using HPLC. The results showed four peaks (P1, P2, P3, and P4), which included chloroform and ethyl acetate fractions (Figures 4(a)-4(b)). The two main peaks, P2 and P4, were associated with arctiin and arctigenin, respectively. We then investigated the effects of arctiin and arctigenin on intestinal barrier function and OVA permeation across Caco-2 cell monolayers. Arctigenin, not arctiin, enhanced the TEER value and inhibited OVA permeation (Figures 4(c) and 4(d)). Moreover, the TEER increased and the OVA flux decreased in a dose-dependent manner, in cells incubated with increasing concentrations (1–200 *μ*mol/L) of arctigenin (Figures 4(e) and 4(f)). Thus, we suggest that arctigenin is an active component of* F. Arctii* and enhances the intestinal barrier function and suppresses OVA permeation across Caco-2 cell monolayers.

## 4. Discussion

Arctigenin and arctiin are lignans found in many plants of the Asteraceae family, and their physiological functions have been identified by researchers. Typical functions of arctiin include protective [14], anti-inflammatory [15], and antidiabetic [16] effects, whereas those of arctigenin were anti-inflammatory [17], anti-cancer [18], and neuroprotective [19] effects. In the present study, we showed that arctigenin from* F. Arctii* enhanced the intestinal barrier function in human intestinal epithelial Caco-2 cells, but arctiin has no such effect. Both arctigenin and arctiin were well-known active components of* F. Arctii*. Furthermore, arctiin is the glycoside of arctigenin. Nevertheless, they have different physiological and pharmacological functions and mechanisms. For example, arctigenin effectively inhibited intestinal inflammation (body weight loss, proinflammatory cytokines, and crypt destruction) by suppressing the activation of mitogen-activated protein kinases and NF-*κ*B in dextran sulfate sodium-induced colitis model [20]. However, arctiin did not significantly ameliorate dextran sulfate sodium-induced symptoms such as body weight loss, inflammatory index, colon length, and myeloperoxidase activity. Our results also showed different functions of arctigenin and arctiin on intestinal barrier function. Therefore, although arctigenin is an aglycon of arctiin, the physiological effects of arctigenin differ with arctiin.

Recently, the major components of* F. Arctii* have been identified, such as chlorogenic acid, arctiin, and arctigenin [21]. Besides these components, it contains many constituents such as dicaffeoylquinic acid, lappaol H, lappaol C, arctignan E, matairesinol, lappaol A, matairesinoside, lappaol B, diarctigenin, and methyl arctate-b.

Our result showed that arctigenin from* F. Arctii* enhanced intestinal barrier function. However, we suggest that other components also may have the potential on enhancement of intestinal barrier function. For example, chlorogenic acid decreased intestinal permeability through increasing intestinal expression of tight junction proteins such as occludin and zonula occludens-1 in weaned rats challenged with lipopolysaccharide [22]. Therefore, we will investigate the effects of other components from* F. Arctii* on intestinal barrier function in further study.

## 5. Conclusions

In summary,* F. Arctii* enhanced intestinal barrier function in human intestinal epithelial Caco-2 cells. Active component of the extract was arctigenin. We expect that the reinforcing effect of arctigenin from* F. Arctii* could contribute to prevention of inflammatory, allergic, and infectious diseases.

## Figures and Tables

**Table 1:** Yield (%, w/w) and total phenol content (mg/g) of fractions isolated from *Fructus Arctii* extract.

FAE	Yield	Total phenols content
(*Fructus Arctii*)	(%, w/w)	(mg/g)
Hexane fraction	39.98	16.25 ± 0.9
Chloroform fraction	41	237.44 ± 2.46
Ethyl acetate fraction	7.89	412.78 ± 10.47
Butanol fraction	5.66	274.79 ± 1.32
Water fraction	5.47	84.62 ± 0.16

## Abbreviations

DMEM: Dulbecco's modified Eagle's medium
FAE: * Fructus Arctii* extract
FBS: Fetal bovine serum
HBSS: Hank's balanced salt solution
HPLC: High-performance liquid chromatography
HRP: Horseradish peroxidase
NEAA: Penicillin-streptomycin, nonessential amino acids
NF-*κ*B: Nuclear factor kappa B
OVA: Ovalbumin
PBS: Phosphate-buffered saline
TEER: Transepithelial electrical resistance
TJ: Tight junction
TPC: Total phenolic content.
